# Specific features of ß-catenin-mutated hepatocellular carcinomas

**DOI:** 10.1038/s41416-024-02849-7

**Published:** 2024-09-11

**Authors:** Camille Dantzer, Lydia Dif, Justine Vaché, Sara Basbous, Clotilde Billottet, Violaine Moreau

**Affiliations:** University Bordeaux, INSERM, BRIC, U1312 Bordeaux, France

**Keywords:** Liver cancer, Oncogenes

## Abstract

*CTNNB1*, encoding the ß-catenin protein, is a key oncogene contributing to liver carcinogenesis. Hepatocellular carcinoma (HCC) is the most common form of primary liver cancer in adult, representing the third leading cause of cancer-related death. Aberrant activation of the Wnt/ß-catenin pathway, mainly due to mutations of the *CTNNB1* gene, is observed in a significant subset of HCC. In this review, we first resume the major recent advances in HCC classification with a focus on *CTNNB1*-mutated HCC subclass. We present the regulatory mechanisms involved in β-catenin stabilisation, transcriptional activity and binding to partner proteins. We then describe specific phenotypic characteristics of *CTNNB1*-mutated HCC thanks to their unique gene expression patterns. *CTNNB1*-mutated HCC constitute a full-fledged subclass of HCC with distinct pathological features such as well-differentiated cells with low proliferation rate, association to cholestasis, metabolic alterations, immune exclusion and invasion. Finally, we discuss therapeutic approaches to target ß-catenin-mutated liver tumours and innovative perspectives for future drug developments.

## Introduction

Hepatocellular carcinoma (HCC) is the major type of primary liver cancer in adult. It is the 6th most frequent cancer worldwide and the 3rd leading cause of cancer-related death [1]. HCC is a heterogeneous disease with a variety of etiological factors including hepatitis B virus (HBV), hepatitis C virus (HCV), alcoholic cirrhosis and metabolic dysfunction-associated steatohepatitis (MASH). Late diagnosis and the lack of efficient therapies account for the poor prognosis of HCC. These last years, the treatment of advanced HCC has undergone a therapeutic revolution with the emergence of immune-checkpoint inhibitors. The combination of Atezolizumab (anti-PD-L1 antibody) and Bevacizumab (anti-VEGF antibody) became the first-line FDA-approved therapy [2]. However, only a subset of patients responds to these treatments [3]. The development of efficient and targeted therapies requires molecular analyzes to understand HCC diversity. Among the pathways found implicated in liver carcinogenesis, the activation of the Wnt/ß-catenin pathway, mainly due to mutations in the *CTNNB1* gene encoding ß-catenin protein, is observed in a significant subset of HCC. *CTNNB1*-mutated HCC are defined at the molecular, histo-pathological and clinical levels as tumours with specific characteristics, which we aim to summarise herein. We also discuss about therapeutic advances to target ß-catenin mutated liver tumours.

## Classification of HCC

**Fig. 1:**
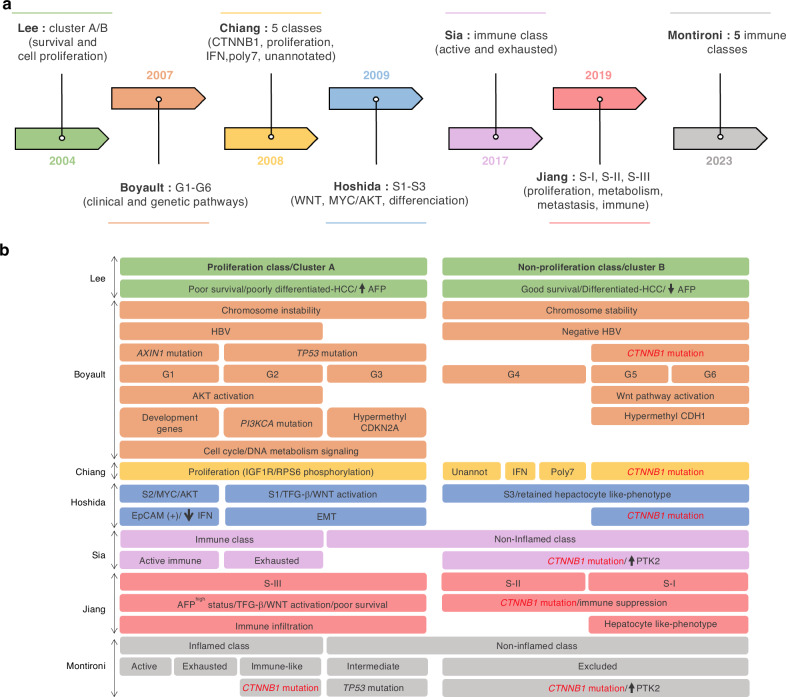
Main molecular classes and subclasses of HCC. **a** HCC classification and timeline and (**b**) features. HCC can be divided in two major groups ‘proliferative’ and ‘non-proliferative’. The proliferation group is mainly characterised by high level of AFP, poor survival, poor differentiated-HCC, chromosome instability, HBV infection, mitogenic/stem-cell like properties and an immunogenic phenotype. The non-proliferative group is characterised by low level of AFP, good survival, differentiated-HCC, chromosome stability, hypermethylation, *CTNNB1* mutation and an immune suppression profile.

Using global unbiased approaches, several groups have proposed HCC molecular classifications mainly based on transcriptomic and more recently on proteomic data [4–9] (Fig. 1a).

HCC have been first divided into two major molecular groups (clusters A and B) based on the proliferation level [4]. The expression of genes involved in cell proliferation is higher in cluster A. Cluster A also contains a higher percentage of AFP+ (Alpha-FoetoProtein) patients and its overall survival time is shorter compared to cluster B (Fig. 1a, b).

Boyault et al. divided HCC into two classes based on chromosome stability status, but further identified 6 groups of HCC (G1-G6) [5]. G1-G3 group tumours are associated with high chromosomal instability and enriched with cell-cycle/proliferation/DNA metabolism genes. G4 group is a heterogenous group of HCC tumours. G5-G6 groups are highly related to *CTNNB1* mutations (Fig. 1a, b).

Chiang et al. described five different classes including ‘CTNNB1’ enriched for *CTNNB1* mutations and ß-catenin nuclear localisation, ‘proliferation’ enriched for IGF1R and RPS6 phosphorylation, ‘interferon (IFN)-related’ associated with smaller tumour size, a new class ‘polysomy of chromosome 7’ and one ‘unannotated’ associated with the focal gains of VEGFA (Fig. 1a, b) [6].

Hoshida et al. reported three distinct HCC molecular classes (S1, S2 and S3) [7]. S1 class exhibits tumours with vascular invasion/satellites lesions and is associated to Wnt/TGF-ß activity and to expression of genes involved in epithelial-mesenchymal transition (EMT). S2 class is characterised by proliferative tumours with a suppression of INF target genes. *CTNNB1*-mutated tumours are grouped into the S3 class that retains more hepatocyte like-phenotype with well-differentiated tumours (Fig. 1a, b).

The Cancer Genome Atlas Research Network (TCGA) conducted the first extensive multi-platforms analysis of HCC and proposed the iCluster Classification divided in three subtypes (iCluster 1-3) [10]. The comprehensive analysis of multiple data platforms combined with clinical data has facilitated the discovery of biological insights, the identification of potential therapeutic targets, and the characterisation of distinct subclasses with prognostic implications.

The quantitative proteomic analyses of Jiang et al. stratified early-stage HCC into three subclasses (S-I, S-II and S-III) [8]. S-III tumours exhibit more aggressive characteristics including the upregulation of proteins associated with a poor prognosis (such as TGFβ1, KRT19 and MMP9) and the activation of tumour-promotion pathways (such as TGFβ, HIF1, integrin and Rho GTPases pathways). The less-aggressive classes were preferentially found in S-I and S-II tumours, consistent with the *CTNNB1* mutations. S-I tumours also exhibited hepatocyte-like characteristics (Fig. 1a, b).

Several immunogenomic classifications of HCC have emerged. Sia et al. firstly divided HCC in two classes: Immune and Non-inflamed. Interestingly, *CTNNB1* mutations were restricted to the Non-inflamed class associated with low immune signature (in particular T cells) (Fig. 1a, b) [11]. More recently, Montironi et al. classified the HCC inflamed class (37%) in three subclasses (Active, Exhausted and Immune-like) and the non-inflamed class (63%) in two subclasses (Intermediate and Excluded) (Fig. 1a, b) [9]. The inflamed class is characterised by high IFN signalling, PD-1 signalling and overexpression of genes related to lymphocyte chemotaxis such as CXCL9 and CXCL10. The Intermediate subclass (43%) presented *TP53* mutations and chromosomal losses. The Excluded subclass (20%) is mainly characterised by *CTNNB1* mutations and *PTK2* overexpression. However, it is important to note that two-thirds of *CTNNB1*-mutated tumours are associated with the Excluded subclass, described as devoid of immune cell infiltrates, while the other third is associated within inflamed class.

During the past two decades, several studies report different HCC molecular classifications due to the heterogeneous and complex disease with various etiological factors. Today a consensus molecular taxonomy of the disease seems to have emerged (Fig. 1b). Importantly, dysregulation of the Wnt pathway occurs in two different subclasses of HCC (Wnt-TGFß and *CTNNB1*-mutated), with clearly different transcriptomic and proteomic profiles and outcome. Thus, altered gene or protein expressions due to ß-catenin mutations define *CTNNB1*-mutated HCC as a full-fledged sub-class of liver tumours with specific features.

## *CTNNB1* is an oncogene in the liver

Wnt/β-Catenin plays a critical role in liver homeostasis and regeneration. β-catenin is one of the key molecules regulating the hepatic zonation pattern characterised by the molecular heterogeneity and location of hepatocytes within the hepatic lobule [12].

**Fig. 2:**
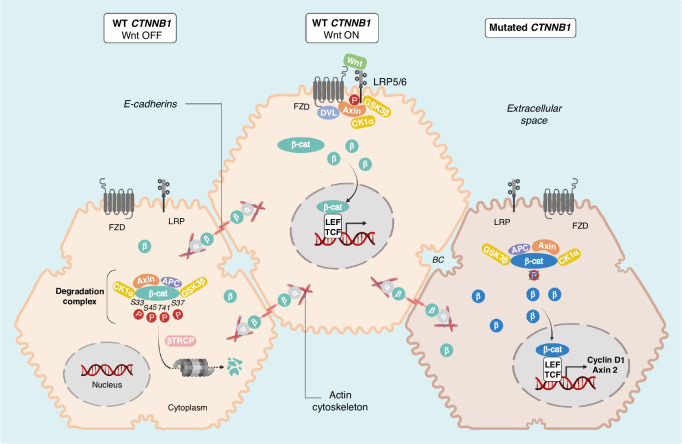
Involvement of wild-type (WT) and mutated ß-catenin in adhesion and Wnt signalling in HCC. β-catenin plays a dual role in hepatocytes: mediator of adhesion and key actor of the Wnt/ß-catenin signalling pathway. When β-catenin proteins are mutated, they are stabilised and translocated into the nucleus to play their role of transcriptional co-factor for genes involved in tumour progression. APC, adenomatous polyposis coli protein; βTRCP, beta-transducin repeat containing; E3 ubiquitin protein ligase; BC, bile canaliculi; CK1α, casein kinase 1 isoform-α; DVL, dishevelled; FZD, frizzled; GSK3β, glycogen synthase kinase 3β; LEF, lymphoid enhancer-binding factor; LRP low-density lipoprotein receptor-related protein; TCF T cell factor. Figure designed with BioRender.

β-catenin is a key switch component of the canonical pathway that plays a dual role in epithelial cells: 1) mediator of adhesion in association with E-cadherin at adherens junctions, where it interacts with α-catenin, thereby indirectly modulating the actin cytoskeleton and 2) transcriptional co-factor of the Wnt/ß-catenin signalling pathway acting in the nucleus (Fig. 2).

### β-catenin structure

**Fig. 3:**
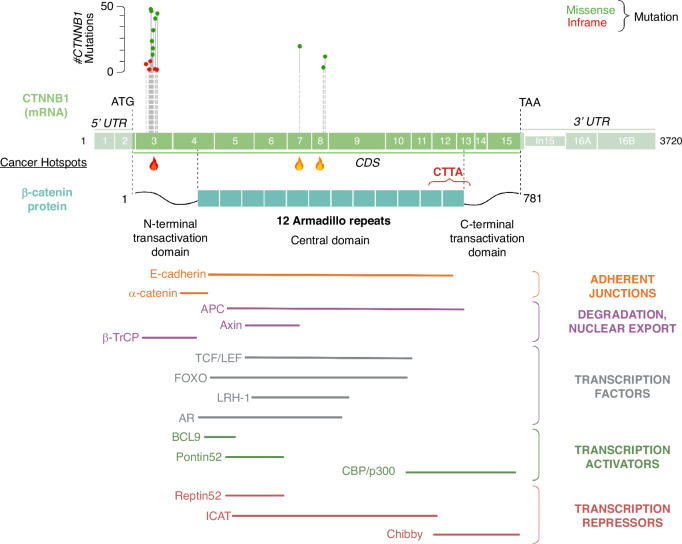
*CTNNB1* mutations in HCC and binding partners of the ß-catenin protein. Mutations of *CTNNB1* are mostly found in the exon 3 but two other hotspots are also located in exons 7 and 8. The β-catenin protein has a central region composed of 12 Armadillo repeats that allows the interaction with many other partners. UTR, untranslated region; CDS, coding sequence; CTTA, C-Terminal Transcriptional Activators; APC, adenomatous polyposis coli protein, βTRCP, beta-transducin repeat containing E3 ubiquitin protein ligase; LEF, lymphoid enhancer-binding factor; TCF, T cell factor; FOXO, forkhead box; LRH-1, liver receptor homolog-1; AR, androgen receptor; BCL9, B-cell lymphoma-9; ICAT, inhibitor of β-catenin and Tcf-4; CBP, CREB-binding protein. Inspired from cBioPortal. Figure designed with BioRender.

β-catenin protein is encoded by the *CTNNB1* gene and belongs to the Armadillo repeat protein superfamily [13]. The N-terminal domain of the β-catenin protein is an important site of post-translation modifications (serine 33 (S33), serine 37 (S37), threonine 41 (T41) and serine 45 (S45)) involving phosphorylation by several kinases such as Glycogen Synthase Kinase 3α and 3β (GSK3α and GSK3β) and Casein Kinase 1α (CK1α). The central domain (residues 141–664) of β-catenin is composed of 12 Armadillo repeats that form a relatively rigid scaffold domain allowing β-catenin to interact with many proteins at the membrane, in the cytosol and in the nucleus, such as E-cadherin, Axin or TCF/LEF. The β-catenin C-terminal domain is composed of a transactivation domain including the CTTA (C-Terminal Transcriptional Activators) domain that binds to many complexes promoting β-catenin-mediated gene transcription (Fig. 3).

### Wnt/ß-catenin pathway: regulation at the physiological state

At basal level, cytoplasmic β-catenin is maintained and subtly controlled by the so-called β-catenin destruction complex that regulates the switch between the Wnt-off and the Wnt-on state of the Wnt/β-catenin pathway. This complex is cytoplasmic and composed of several proteins such as Adenoma Polyposis Coli (APC), Axin, CK1α, GSK3α/β and the E3-ubiquitin ligase β-Transducin repeats-containing protein (β-TrCP) [14–16]. Axin and APC are two scaffold proteins that interact together and bind β-catenin (Figs. 2–3). This core Axin/APC interaction is crucial to tightly control β-catenin level by coordinating sequential phosphorylation of β-catenin N-terminal domain. Both Axin and APC are tumour suppressor genes, and their mutations have also been reported in HCC [17, 18].

The Wnt/β-catenin pathway is composed of a G-protein coupled receptor Frizzled (Frz) and a co-receptor LRP (LDL-Related Protein) at the cell membrane. The Wnt-off state is characterised by the absence of the Wnt ligand allowing the destruction complex to be maintained. β-catenin can be released from the adherent junctions by the activity of protein kinases or by the downregulation of E-cadherin. Free excess of β-catenin is immediately phosphorylated by CK1α (S45) and then by GSK3β (T41, S37 and S33) [14]. β-catenin phosphorylation at S33 and S37 triggers the recruitment of β-TrCP and therefore β-catenin ubiquitination and proteasomal degradation, resulting in a low level of cytoplasmic β-catenin (Fig. 2). A portion of β-catenin protected by APC can also be kept in the cytoplasm.

The presence of the Wnt ligand induces the Wnt-on state that dissociates and blocks the activity of the destruction complex. β-catenin is no longer degraded leading to its cytoplasmic accumulation and translocation into the nucleus. In the nucleus, β-catenin plays its role as a transcriptional co-factor and associates with transcription factors such as TCF/LEF family factors and drives the transcription of several β-catenin target genes (Fig. 2). This transcriptional activity of β-catenin induces the expression of genes involved in physiological and pathological processes such as embryonic development and tumour progression [19, 20]. ß-catenin target genes include canonical Wnt targets such as *AXIN2* but also liver-specific targets such as *GLUL* encoding glutamine synthetase (GS) that play important roles in the features of β-catenin-mutated HCC (Supplemental Table I).

The Wnt/β-catenin pathway can also be regulated by others proteins such as Lgr5 and R-spondin1. Lgr5 is a stem cell marker that associates with the Frz/LRP receptors complex and the binding of soluble R-spondin1 to Lgr5 mediates activation of the Wnt pathway [21]. Lgr5 is not expressed in the homeostatic adult liver. But, by using organoids, Huch et al. showed that a population of Lgr5^+^ liver stem cells actively contributed to liver regeneration via de novo generation of hepatocytes and ductal cells [22].

### Wnt/ß-catenin pathway: regulation at the pathological state

*CTNNB1* was identified as one of the key oncogenes involved in HCC. Constitutively active β-catenin mutations, leading to its uncontrolled transcriptional activity, are present in 30% to 40% of HCC patients. Mutations are mostly found in the exon 3 of *CTNNB1* gene and are heterozygous in 60% of HCC [23] (Fig. 3). The region encoded by exon 3 contains the phosphorylation sites that physiologically lead to the ubiquitination and proteasomal degradation of β-catenin. β-catenin mutations at, or surrounding, the above-mentioned serine and threonine residues, are often missenses or deletions generating mutated forms of β-catenin that escape degradation. Thus, mutated β-catenin proteins are stabilised, translocate into the nucleus where they act as an uncontrolled transcriptional co-factor. Two other hotspots of mutations, in exon 7 at lysine residue 335 (K335) and in exon 8 at asparagine residue 387 (N387), were also discovered in β-catenin-mutated HCC [24]. However, nuclear β-catenin is not always a reliable indicator of *CTNNB1* mutation status in HCC. Activation of the Wnt/β-catenin pathway in HCC has been also linked to HBV infection, *APC* and *AXIN1* mutations [17, 18, 25, 26]. *CTNNB1* and *AXIN1* mutations are mutually exclusive in human HCC and recently Abitbol et al. reported that a subset of AXIN1-mutated HCC are highly enriched in Notch and Hippo pathways that induce expression of genes associated with invasion, stemness and poor prognosis [27].

### ß-catenin-mediated liver carcinogenesis in mouse models

The oncogenic role of β-catenin has been studied in different HCC mouse models. First it was demonstrated that chemically induced HCC showed high prevalence of ß-catenin mutations. Whereas N-nitrosodiethylamine (DEN) carcinogen induced liver tumours without detectable ß-catenin mutations, the addition of phenobarbital as a tumour promoter induced a strong selective pressure allowing the emergence of *CTNNB1*-mutated HCC in about 80% of mice [28]. By using genetic lineage tracing strategies, Ang et al. demonstrated that Lgr5+ hepatocytes are susceptible to hepatocarcinogenesis induced by in mice [29]. ß-catenin activation, without mutation, is relatively common in HCC developed in transgenic mice that overexpressed c-myc, c-myc/TGF-ß1 or c-myc/E2F1 in the liver [30, 31]. On the other hand, many studies demonstrated that ß-catenin mutations play a critical role in hepatocarcinogenesis in cooperation with other oncogenes [23, 32]. More recently, by performing hydrodynamic tail-vein injections, ß-catenin involvement in HCC was also addressed through a global approach combining *CTNNB1* mutations with various other driver genes [33]. Besides the cooperation, this raises the question whether *CTNNB1* mutations alone may give rise to liver development in mouse liver. This question remained controversial for decades. Indeed, whereas Harada et al. demonstrated in 2002 that the expression of ∆exon3 *CTNNB1* in mouse hepatocytes was not sufficient for hepatocarcinogenesis [34], Colnot et al. demonstrated that the deletion of *APC* in the liver allows the formation of activating ß-catenin mediated tumours in mice [17]. Through adeno-associated virus vectors-mediated *CTNNB1* exon 3 deletion by CRISPR-based editing in vivo, Colnot’s team further managed to generate liver tumours using the right dosing of ß-catenin activation within hepatocytes [35]. Thus, in both humans and animals, ß-catenin is an oncogene whose activation causes liver tumours.

## Features of *CTNNB1* mutated HCC

**Fig. 4:**
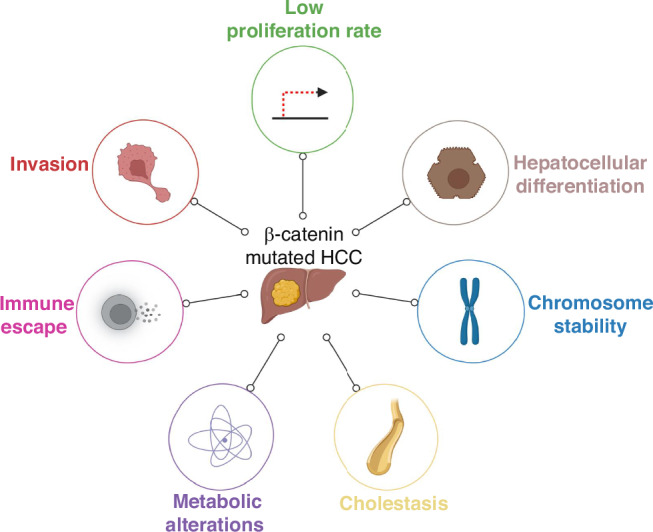
Specific features of ß-catenin-mutated HCC. ß-catenin-mutated HCC display particular intrinsic biological properties. They present a low proliferation rate, important cellular differentiation and chromosome stability. Moreover, these tumours are characterised by cholestasis, metabolic alterations, invasion and capability to escape the immune system. Figure designed with BioRender.

*CTNNB1* mutations define HCC with specific phenotypic features (Fig. 4). These tumours are well differentiated tumours with low proliferation rate and associated to cholestasis, to metabolic alterations, to immune exclusion and to invasion. In this part, we attempt to summarise these features.

### Well-differentiated tumours with low proliferation rate

From a clinical point of view, β-catenin mutated HCC are characterised as tumours with a less aggressive phenotype [7] and a better prognosis than other HCC. These tumours are large with an intact capsule, display microtrabecular and pseudoglandular histological patterns [36–39]. Recently, Torbenson et al. highlighted a histological heterogeneity, notably in the presence or not of pseudoglands, among *CTNNB1*-mutated HCC that could be explained by gender or differential mutation sites in the gene [39]. It is noticeable that some contrasting data exist in advanced HCC, indicating that *CTNNB1* mutations are not linked to prognosis or differentiation grade in a context of HBV infection [40] or in a context of non-nuclear overexpression of mutated β-catenin [41].

*CTNNB1*-mutated tumours were described by several studies as well-differentiated [7, 36, 37]. Audard et al. first showed that tumours with strong GS expression were linked to a well-differentiated pattern in a 202 HCC patients cohort [36]. The uniform immunohistochemical GS expression was clearly associated with β-catenin mutations in HCC allowing their identification [36, 37, 42]. As mentioned before, based on meta-analyses of gene and protein expression profiles, Hoshida et al. and Jiang et al. associated tumours with *CTNNB1* mutations to a hepatocyte-like phenotype [7, 8]. These results were further confirmed by Calderaro et al. that investigated molecular-phenotype correlations in a large series of HCC. β-catenin-mutated HCC were characterised by a low proliferation [37] and chromosome stability, these tumours presenting less genetic alterations than other HCC and thus being less aggressive [5, 38]. At the molecular level, TBX3 was recently involved in these phenomena by Liang et al. They showed that *TBX3* mRNA expression was higher in *CTNNB1*-mutated HCC and associated with an increased differentiation status and less malignancy [43]. LKB1 may also participate to *CTNNB1*-mutated HCC particular phenotype and better prognosis. Charawi et al. described its overexpression and post-transcriptional accumulation in such tumours and suggested that it may act as a tumour suppressor by inhibiting cell proliferation, favoring mitotic integrity and conferring a well-differentiated growth pattern [44].

### Association to cholestasis

The bile is produced and secreted by hepatocytes in the bile canaliculus, which is the first structure of biliary tract. A normal membrane polarity is essential for bile canaliculus formation and hepatocyte function and its loss may lead to many diseases like cholestasis. Cholestasis is a condition where the bile is not properly driven from the liver to the duodenum, i.e. resulting in the accumulation of bile into the hepatic parenchyma. A link between β-catenin and bile canalicular abnormalities is known since the middle of 2000’s but it remains puzzling. On one hand, cholestasis is a key feature of ß-catenin mutated HCC [36] and β-catenin mutations were described to alter both bile transport through the biliary tract and bile synthesis. On the other hand, the liver-specific β-catenin knock-out (KO) mice were shown to also develop intrahepatic cholestasis associated with bile canalicular abnormalities and bile secretory defect [45, 46]. These data suggest that loss of ß-catenin as well as excessive activation of ß-catenin may lead to cholestatic defect in the liver. Whether this phenotype is due to structural or transcriptional activity of ß-catenin is largely unknown. Several studies reported the involvement of cell adhesion molecules in the maintenance of bile canaliculi. In cultured ß-catenin mutated HepG2 cells or embryonic chicken hepatocytes, E-cadherin was shown to be important for bile canaliculi lumen extension [47, 48]. However, its liver specific KO in mice did not alter hepatocyte polarity and bile canaliculus formation [49]. We recently described an in vitro approach that allows uncoupling the functions of Wild-Type (WT) or mutated forms of β-catenin [50]. We found that, in HepG2 cells, mutated ß-catenin decreases the number, the size, and the functionality of bile canaliculi, whereas WT ß-catenin is important for their maintenance. Thus, as demonstrated for the development of bile ducts [51], ß-catenin has to be kept at the right level as loss of ß-catenin or ß-catenin overactivation is detrimental for bile canaliculi, formation and/or stabilisation. Increasing evidence also indicates that the Wnt/β-catenin pathway participates in the regulation of bile synthesis. Bile acid synthesis is largely regulated by mechanisms involving the Farnesoid X receptor (FXR) and depends on the rate-limiting enzyme CYP7A1. In this line, expression of oncogenic ß-catenin in mouse hepatocytes was described to lead to the development of severe cholestasis with alteration of expression of CYP7A1 [52]. Using the APC-KO mouse model mimicking ß-catenin dependent tumorigenesis, cholestasis was also shown to be mediated by the up-regulation of CYP7A1 and of CYP27A1 which are direct targets of ß-catenin involved in bile acid synthesis [53].

### Association to metabolic alterations

Reprogramming cellular metabolism is a core hallmark of cancer initiation and progression. Deregulations of well-known metabolic pathways such as glucose, TriCarboxylic Acid (TCA) cycle, fatty acid β-oxidation (FAO) and amino acid metabolisms are emphasised in the pathogenesis of HCC [54]. The aerobic glycolytic pathway, known as the Warburg effect, has been considered as the key metabolic pathway involved in cancer cells [55] and is reported to confer immune escape properties to cancer cells [56]. The Warburg effect is characterised by the use of glycolysis with elevated glucose uptake and lactate production, instead of mitochondrial oxidative phosphorylation. Using a combined tissue transcriptomic and metabolomic approach, Beyoglu et al. showed no differences in glucose uptake and lactate production in G5-G6 groups suggesting that glucose metabolism remodelling does not occur in β-catenin mutated HCC [57]. This is consistent with in vivo studies showing that no increase in lactate production was observed in mice bearing β-catenin-mutated liver tumours [58, 59]. This was also confirmed by Senni et al. who showed that murine β-catenin activation-mediated HCC are not glycolytic [60].

β-catenin regulates the expression of acetaldehyde dehydrogenases (e.g., ALDH2, ALDH3A1, and ALDH3A2) thus controlling TCA cycle and FAO [61], two pathways involved in hepatic mitochondrial homeostasis that are extremely relevant energy sources for the proliferation of tumour cells [62–64]. Senni et al. found that mutated β-catenin leads to the induction of FAO in HCC and showed that the treatment with etomoxir (a FAO inhibitor) induced an arrest of HCC progression [60]. Regarding amino acid metabolism, glutamine has been considered as an important amino acid donor of metabolic intermediates (carbon and nitrogen) to fuel biosynthesis. Numerous target genes of β-catenin are involved in glutamine metabolism such as *GLUL* encoding GS, glutamate transporter *SLC1A2* and ornithine aminotransferase *OAT* and the regulation of these three genes by β-catenin has been reported as a contributing factor to liver carcinogenesis [65]. *GLUL* is involved in ammonia detoxification [66] and recently Dai et al. showed that genetic ablation of hepatic *GLUL* accelerated HCC development in activated-β-catenin mice [67]. Mechanistically, *GLUL* deletion exacerbated hyperammonemia and facilitated the production of glutamate-derived nonessential amino acids, which further stimulated the mammalian target of rapamycin complex 1 (mTORC1). The level of *GLUL* expression is often high in mutated-β-catenin HCC patients, whereas for a subset of HCC patients *GLUL* is low with a strong activation of mTORC1. Choline is also an essential nutrient that supports lipid metabolism, lipid transport and is the major methyl-group donor in cells leading to changes in gene expression. Gougelet et al. showed that ß-catenin-mutated HCC exhibit an accumulation of choline-derived products and that a choline-deficient diet induces regression of β-catenin-mutated HCC in mice [68].

### Immune exclusion

As previously described, HCC tumours immunologically classified as ‘Excluded’ show enrichment of *CTNNB1* mutations and are described as cold tumours with regard to density of immune cell infiltration [9]. The tumour immune microenvironment analysis of this “Excluded subclass” showed an important immune depletion including CD8 + T cells, B cells, M1-type macrophages and dendritic cells (DC), associated to a resistance to immunotherapy [9, 69]. Using genetically engineered-mouse models of HCC, ß-catenin activation was described to promote immune escape, i.e. defective recruitment of DC and consequently impaired T cell activity, leading to anti-PD-1 therapy resistance [70]. These tumours express less CCL5 chemokine and other major cytokines and chemokines involved in lymphocyte chemotaxis such as CXCL10. Interestingly, the re-expression of CCL5 restored immune surveillance in these mice. Wnt/ß-catenin signalling has also been involved in immune evasion in human and mouse cultured cells. Intrinsic activation of β-catenin in a cellular model engineered by CRISPR/Cas9 was found to downregulate CCL20 and CXCL2 cytokine secretion and induce immune evasion in mouse xenografts [71]. Recently, our team showed for the first time that, in HCC, mutation or activation of β-catenin promotes a decrease in immune cell infiltration through a default in exosome secretion [72]. We have identified the ß-catenin-dependent decrease in expression of *RAB27A* and *SDC4* genes, two key players in the exosomal machinery. However, few studies reported that the mutational status of *CTNNB1* is not a dominant feature to predict resistance to immune-checkpoint inhibitors [73, 74]. As previously described, one third of β-catenin-mutated HCC belong to the Inflamed class which is characterised by IFN-signalling and active antigen-presenting machinery [9]. These data indicate that in β-catenin-activated tumours, the immune environment is heterogeneous, with the immune response possibly influenced by a shift in balance between the immunosuppressive effects of Wnt/β-catenin and the pro-inflammatory IFN pathway. Distinguishing between these profiles may help elucidate the discrepancies noted in the predictive significance of *CTNNB1* mutations in HCC.

### Association to invasion

As described above, ß-catenin-mutated HCC are described as well-differentiated tumours, less aggressive and of better prognosis, when compared to non-mutated HCC. However, invasive criteria, such as satellite nodules or perivascular invasion, and EMT markers were often associated to ß-catenin activation in liver tumours. Indeed, a meta-analysis regrouping 22 studies and 2334 human HCC cases found that aberrant cytoplasmic and nuclear β-catenin expression is significantly increased and closely associated with metastasis and vascular invasion [75]. Higher incidence of vascular invasion and greater tumour size have been also observed in the *CTNNB1* mutated group of HCC cases [76]. Moreover, the G6 group is characterised by the downregulation of E-cadherin expression and the high invasive potential indicated by the presence of satellite nodules around the principal tumour [5]. In the c-myc/TGF-α transgenic mouse model, activation of ß-catenin through phenobarbital treatment was shown to provide an invasive advantage to the tumours, where a cytoplasmic relocalisation of E-cadherin was observed [77]. Matrix metalloproteinases (MMPs) also contribute to the invasive properties of HCC cells and MMP-9 is a target of β-catenin signalling that may regulate HCC progression as suggested in vitro and in vivo studies [78, 79]. Thus, ß-catenin-mutated tumour cells are associated with an increase capability of migration and invasion, and ß-catenin-mutated HCC with loco-regional invasive features. To reconcile these statements with the fact that these tumours were also described as having a better prognosis, comprehensive molecular analyses of human metastatic HCC were lacking. Very recently, Sun et al. provided a dissection of the complex evolutionary process of metastasis, thanks to the multi-omic profiling of 257 primary and 176 metastatic regions from 182 HCC patients [80]. They found that genes involved in the Wnt oncogenic pathway, such as *CTNNB1*, were not selected subclonal driver mutations during progression to metastasis in distant organs, probably due to a microenvironment low in activated fibroblasts required for a pro-metastatic phenotype.

## Therapeutic approaches to target ß-catenin

### ß-catenin targeted therapies

Due to its strong involvement in carcinogenesis, multiple strategies to target β-catenin pathway appeared. Several molecules have even been the subject of clinical trials for Wnt/ß-catenin-activated tumours [81]. Herein we will focus on drugs that show efficiency and interest for ß-catenin-mutated liver cancers.

The literature is rich of compounds targeting the Wnt ligand and the receptor Frizzled or different actors impacting this pathway [82]. These compounds although effective in stopping cell proliferation, create a high toxicity in normal cells. Moreover, as *exon3 CTNNB1* mutations result in ligand-independent pathway activation, targeting strategies at Wnt/Frizzled level are not suitable.

β-catenin is a multi-partner protein, therefore several strategies targeting β-catenin protein-protein interactions were generated. The main well-described interaction occurs in the nucleus with TCF/LEF transcription factors. Molecules that prevent the interaction with TCF4 were designed but due to several issues (overlap with Axin1 and APC binding sites, domain of interaction with TCF4 very large), this lead to counterproductive effort [83]. p300 or CREB-binding protein (CBP) are also great partners of β-catenin that function as co-activators of TCF/ß-catenin complex, leading to histone acetylation and gene activation. Several drugs, such as ICG-001 or E7386, were identified to inhibit CBP/ß-catenin interaction [84, 85].

ICG-001 was found to inhibit CBP-mediated stemness state thus favoring p300 binding and cell differentiation. For HCC, administration of ICG-001 enhanced the anti-tumour activity of sorafenib in HCC cell lines and in Huh7 cell mouse xenograft model [86]. Moreover, a synergistic effect of ICG-001 with radiotherapy was demonstrated [87]. Interestingly, the addition of ICG-001 to radiotherapy exhibited better anti-tumour and survival-prolong efficacy in C57BL/6 than nude mice. Analyses revealed that ICG-001 plus radiotherapy boosted the infiltration CD8 + T cells, and reduced the number of Tregs in tumours [87]. More recently, combined application of ICG-001 and immune checkpoint inhibitors exhibits significantly enhanced antitumor efficacy. The rationale behind the combination was to reverse the resistance to immunotherapy by reactivating the infiltration of immune cells through inhibition of ß-catenin. This combination treatment ICG-001/anti-PD-1 effectively suppresses HCC growth in mice by upregulating the expression of CCL5, and promoting the infiltration of immune cells such as DC and CD8 + T cells in the tumour microenvironment [88].

PRI-724 (and its active form C-82) is an advanced derivative of ICG-001, designed to improve pharmacokinetic properties and efficacy. PRI-724 has been studied extensively in preclinical models for various cancers, including colorectal cancer, pancreatic cancer, and haematologic malignancies. Similarly to ICG-001, PRI-724 (C-82) could inhibit liver cancer cell growth. PRI-724 was shown to induce G0/G1 cell cycle arrest and enhance the apoptosis of ß-catenin-mutated cell lines (HepG2 and Huh6) [89]. PRI-724 has progressed through early-phase clinical trials, primarily focusing on safety, tolerability, and preliminary efficacy in advanced solid tumours and fibrotic diseases (Supplemental Table II). Results from these trials indicate that PRI-724 is generally well-tolerated and may offer therapeutic benefits for patients with liver fibrosis. The study evaluating PRI-724 in patients with advanced solid tumours had to be terminated due to low patient enrollment (NCT01302405). Trials with combined treatment were performed for other cancer types such as leukaemia, pancreatic and colon cancers (Supplemental Table II).

E7386 is an improved version of C-82, the active form of PRI-724, in terms of membrane permeability and solubility. E7386 showed antitumor activity in an APC-mutated ECC10 human gastric xenograft model [85]. Even if the mechanism of action of E7386 remains to be clarified [90], this drug is currently in clinical trials for solid tumours, including HCC (Supplemental table II). It is tested now in a phase Ib or II trials in combination with Lenvatinib or with Pembroluzimab (anti-PD-1).

Recently, Tegavivint entered clinical trials. Tegavivint, also known as BC-2059 and identified from a screen for inhibitors of Wnt/b-catenin signalling [91], selectively interferes with the interaction between transducin b-like protein 1 (TBL1) and ß-catenin [92]. Disruption of this complex inhibits ß-catenin nuclear translocation and promotes its degradation. Tegavivint showed antitumor activity preclinically against B-cell lymphoma [93], multiple myeloma [94] and adrenocortical carcinoma [95]. Based on these encouraging results, Tegavivint is currently under different stages of clinical development for the treatment of cancers (Supplemental Table II). For HCC, an ongoing trial is testing Tegavivint monotherapy versus combination with pembrolizumab in patients (NCT05797805).

### Perspectives for future drug development

β-catenin was once considered as an undruggable protein for various reasons. Indeed, it is an ubiquitous protein that, if targeted, generates a lot of toxicity in healthy tissues and a transcriptional co-factor with a flat surface lacking structural pockets that favour drug design.

The central domain of β-catenin composed of Armadillo repeats was the main domain used for all rational drug design, N-terminal and C-terminal domains that are more unstructured were neglected. However, these two domains are highly implicated in phosphorylation, stabilisation and degradation of the protein. Thanks to advancing techniques, new molecules will be designed to target these regions and therefore decrease the specificity issues. Especially molecules that could target the mutated forms of β-catenin, as was the case for KRAS mutants that were considered undruggable for 40 years until specific KrasG12C inhibitors were developed and entered clinical trials [96]. Very recently, WNTinib proved to selectively antagonise the mutated β-catenin pathway and demonstrated unprecedented efficacy both in HCC cellular models and in vivo. This result paves the way to highly precise medicine [97].

Another very promising strategy is to induce β-catenin degradation. MSAB (Methyl Sulfonyl Amino Benzoate), identified from a high throughput screen using a Wnt reporter gene assay in HCT116 cells was found to directly bind to β-catenin and induces its proteasomal degradation [98]. Using the same idea, ProTaC (Proteolysis-Targeting Chimeras) were developed to bind a protein of interest to an E3 ubiquitin ligase, thus leading to its ubiquitination and proteasome degradation. This technique might be useful to deplete the excess of β-catenin in tumour cells although it may not exclude unwanted toxicity. xStax-VHLL is a ProTaC targeting β-catenin, it reduces β-catenin levels in vitro and in vivo in colon cancer inhibiting tumorigenic effects [99].

Molecule vectorisation with nanoparticles is an interesting method to potentialize the effect of drugs on target cells. Nanoparticles are small cargos measuring 10–100 nm in diameter. They optimise the delivery of different molecules such as drugs and nucleic acids to target sites. They can be functionalized with surface proteins to avoid off-target toxicities or increase cellular uptake [100]. Tankyrase inhibitor XAV939 was encapsulated in mannose nanoparticles and showed an immunogenic effect on the tumour microenvironment of immunosuppressive melanoma by inhibiting β-catenin in macrophages [101]. Nanoparticles can deliver multiple cargos such as siRNA. One specific nanoparticle approach was developed to enhance β-catenin destruction complex via the delivery of one siRNA inhibiting LncRNA AFAP1‐AS1, this sensitises breast cancer cells in vitro and in vivo to radiotherapy [102].

Regarding the existing literature on how to target β-catenin and the advances that are made each year in this field, we are about to say that β-catenin is no longer an “undruggable protein”. Researchers now realise that designing a drug to target a ubiquitous protein is no longer the strategy to adapt against cancer. We need precise medicine and by this we mean targeting the mutated form of β-catenin in the cancer tissue without disrupting the healthy cells. Vectorized strategies are very promising but other strategies might emerge and overcome β-catenin unarguability.

## Supplementary information

Supplemental Table legends

Supplemental Table I

Supplemental Table II
