# Supplementary material for: Specific features of ß-catenin-mutated hepatocellular carcinomas
Source: Br J Cancer. 2024 Sep 11;131(12):1871–80. doi: 10.1038/s41416-024-02849-7 (PMC11628615; doi:10.1038/s41416-024-02849-7)
Supplement: Supplementary file 1 — Supplemental Table legends [file 41416_2024_2849_MOESM1_ESM.docx]

***Supplemental table* *legends***

**Supplemental Table I: Listing of ubiquitous and hepatic ß-catenin target genes.** This table groups together some of the classical ß-catenin targets and liver-specific ß -catenin targets.

**Supplemental Table II: Clinical trials using anti-ß-catenin strategies.**
