# Supplementary material for: Specific features of ß-catenin-mutated hepatocellular carcinomas
Source: Br J Cancer. 2024 Sep 11;131(12):1871–80. doi: 10.1038/s41416-024-02849-7 (PMC11628615; doi:10.1038/s41416-024-02849-7)
Supplement: Supplementary file 2 — Supplemental Table I [file 41416_2024_2849_MOESM2_ESM.docx]

**Supplemental Table I: Listing of ubiquitous and hepatic ß-catenin target genes**

The genes below represent a non-exhaustive list of ß-catenin transcriptional targets, dysregulated or not in liver cancers.

| **Gene symbol** | **Gene name** | **Positive/Negative?**  **(References)** | | **Dysregulated in ß-cat mutated liver cancers (references)** |
| --- | --- | --- | --- | --- |
| *Ubiquitous targets* |  |  |  |  |
| *ATF3* | Activating transcription factor 3 | Positive | (1) |  |
| *AXIN2* | Axin 2 | Positive | (2) | (3) |
| *BAMBI* | BMP and activin membrane-bound inhibitor homolog (*Xenopus laevis*) | Positive | (4) | (5) |
| *BIRC5* | Baculoviral IAP repeat-containing 5 (Survivin) | Positive | (6) | (7) |
| *CCND1* | Cyclin D1 | Positive | (8) | (9) |
| *DKK1* | Dickkopf 1 homolog (*Xenopus laevis*) | Positive | (10) | (11)(12) |
| *DUSP6* | Dual specificity phosphatase 6 | Positive | (13) |  |
| *DUSP14* | Dual specificity phosphatase 14 | Positive | (13) |  |
| *EPCAM* | Epithelial cell adhesion molecule | Positive | (14) | (14) |
| *FSCN1* | Fascin1 | Positive | (15) | (16) |
| *HES1* | Hairy and enhancer of split 1 | Negative | (17) |  |
| *IL10* | Interleukin 10 | Positive | (18) |  |
| *LEF1* | Lymphoid enhancer-binding factor 1 | Positive | (19)(20) |  |
| *LGR5* | Leucine-rich repeat containing G protein-coupled receptor 5 | Positive | (21) | (21) |
| *MYC* | c-Myc myelocytomatosis viral oncogene homolog | Positive | (22) |  |
| *NKD1* | Naked cuticle homolog 1 (Drosophila) | Positive | (23) | (23) |
| *NOTUM* | Notum pectinacetylesterase homolog (Drosophila) | Positive | (24) | (24) |
| *PRC1* | Protein regulator of cytokinesis 1 | Positive | (25) | (25) |
| *TGFA* | Transforming growth factor, alpha | Positive | (26) | (26) |
| *SOX9* | SRY (sex determining region Y)-box 9 | Positive | (27) | (28) (29) |
| *Hepatic targets* |  |  |  |  |
| *ALDH (2, 3A1, 3A2)* | Acetaldehyde dehydrogenases | Positive | (30) |  |
| *ARG1* | Arginase, liver | Negative | (31) | (32) |
| *CPS1* | Carbamoyl-phosphate synthetase 1 mitochondrial | Negative | (31) |  |
| *CYP1A1* | Cytochrome P450, family 1, subfamily A, polypeptide 1 | Negative | (33) |  |
| *CYP2E1* | Cytochrome P450, family 2, subfamily E, polypeptide 1 | Negative | (33) |  |
| *CYP27A1* | Cytochrome P450 family 27 subfamily A member 1 | Positive | (34) | (34) |
| *GLT1/SLC1A2* | Glutamate transporter, Solute carrier family 1, member 2 | Positive | (35) | (35) |
| *GLUL* | Glutamate-ammonia ligase (glutamine synthase) | Positive | (35) | (35) |
| *GLS2* | Glutaminase 2 (liver, mitochondrial) | Negative | (31) |  |
| *HDAC2* | Histone deacetylase 2 | Positive | (31) |  |
| *LECT2* | Leukocyte Cell-Derived Chemotaxin 2 | Positive | (36) | (37) |
| *OAT* | Ornithine aminotransferase | Positive | (35) | (35) |
| *LKB1* | liver kinase B1 | Positive | (38) | (38) |
| *REG3A* | Regenerating islet-derived 3 alpha | Positive | (39) |  |
| *RHBG* | Rhesus blood group, B glycoprotein | Positive | (31) |  |
| *RNF43* | Ring finger protein 43 | Positive | (40) |  |
| *TBX3* | T-box 3 | Positive | (41) | (41) |
| *SLC13A3* | Solute carrier family 13, member 3 | Positive | (40) |  |

1. Yaguchi T, Goto Y, Kido K, Mochimaru H, Sakurai T, Tsukamoto N, et al. Immune suppression and resistance mediated by constitutive activation of Wnt/β-catenin signaling in human melanoma cells. J Immunol. 1 sept 2012;189(5):2110‑7.

2. Yan D, Wiesmann M, Rohan M, Chan V, Jefferson AB, Guo L, et al. Elevated expression of axin2 and hnkd mRNA provides evidence that Wnt/beta -catenin signaling is activated in human colon tumors. Proc Natl Acad Sci U S A. 18 déc 2001;98(26):14973‑8.

3. Ishizaki Y, Ikeda S, Fujimori M, Shimizu Y, Kurihara T, Itamoto T, et al. Immunohistochemical analysis and mutational analyses of beta-catenin, Axin family and APC genes in hepatocellular carcinomas. Int J Oncol. mai 2004;24(5):1077‑83.

4. Sekiya T, Adachi S, Kohu K, Yamada T, Higuchi O, Furukawa Y, et al. Identification of BMP and activin membrane-bound inhibitor (BAMBI), an inhibitor of transforming growth factor-beta signaling, as a target of the beta-catenin pathway in colorectal tumor cells. J Biol Chem. 20 févr 2004;279(8):6840‑6.

5. Lee S, Lee MJ, Zhang J, Yu GR, Kim DG. C-terminal-truncated HBV X promotes hepato-oncogenesis through inhibition of tumor-suppressive β-catenin/BAMBI signaling. Exp Mol Med. 2 déc 2016;48(12):e275.

6. Kim PJ, Plescia J, Clevers H, Fearon ER, Altieri DC. Survivin and molecular pathogenesis of colorectal cancer. Lancet. 19 juill 2003;362(9379):205‑9.

7. Peroukides S, Bravou V, Alexopoulos A, Varakis J, Kalofonos H, Papadaki H. Survivin overexpression in HCC and liver cirrhosis differentially correlates with p-STAT3 and E-cadherin. Histol Histopathol. mars 2010;25(3):299‑307.

8. Tetsu O, McCormick F. Beta-catenin regulates expression of cyclin D1 in colon carcinoma cells. Nature. 1 avr 1999;398(6726):422‑6.

9. Patil MA, Lee SA, Macias E, Lam ET, Xu C, Jones KD, et al. Role of cyclin D1 as a mediator of c-Met- and beta-catenin-induced hepatocarcinogenesis. Cancer Res. 1 janv 2009;69(1):253‑61.

10. Niida A, Hiroko T, Kasai M, Furukawa Y, Nakamura Y, Suzuki Y, et al. DKK1, a negative regulator of Wnt signaling, is a target of the beta-catenin/TCF pathway. Oncogene. 4 nov 2004;23(52):8520‑6.

11. Patil MA, Chua MS, Pan KH, Lin R, Lih CJ, Cheung ST, et al. An integrated data analysis approach to characterize genes highly expressed in hepatocellular carcinoma. Oncogene. 26 mai 2005;24(23):3737‑47.

12. Wirths O, Waha A, Weggen S, Schirmacher P, Kühne T, Goodyer CG, et al. Overexpression of human Dickkopf-1, an antagonist of wingless/WNT signaling, in human hepatoblastomas and Wilms’ tumors. Lab Invest. mars 2003;83(3):429‑34.

13. Zeller E, Mock K, Horn M, Colnot S, Schwarz M, Braeuning A. Dual-specificity phosphatases are targets of the Wnt/β-catenin pathway and candidate mediators of β-catenin/Ras signaling interactions. Biol Chem. oct 2012;393(10):1183‑91.

14. Yamashita T, Budhu A, Forgues M, Wang XW. Activation of hepatic stem cell marker EpCAM by Wnt-beta-catenin signaling in hepatocellular carcinoma. Cancer Res. 15 nov 2007;67(22):10831‑9.

15. Vignjevic D, Schoumacher M, Gavert N, Janssen KP, Jih G, Lae M, et al. Fascin, a novel target of beta-catenin-TCF signaling, is expressed at the invasive front of human colon cancer. Cancer Res. 15 juill 2007;67(14):6844‑53.

16. Gest C, Sena S, Dif L, Neaud V, Loesch R, Dugot-Senant N, et al. Antagonism between wild-type and mutant β-catenin controls hepatoblastoma differentiation via fascin-1. JHEP Rep. mai 2023;5(5):100691.

17. Peignon G, Durand A, Cacheux W, Ayrault O, Terris B, Laurent-Puig P, et al. Complex interplay between β-catenin signalling and Notch effectors in intestinal tumorigenesis. Gut. févr 2011;60(2):166‑76.

18. Spranger S, Bao R, Gajewski TF. Melanoma-intrinsic beta-catenin signalling prevents anti-tumour immunity. Nature. 9 juill 2015;523(7559):231‑5.

19. Hovanes K, Li TW, Munguia JE, Truong T, Milovanovic T, Lawrence Marsh J, et al. Beta-catenin-sensitive isoforms of lymphoid enhancer factor-1 are selectively expressed in colon cancer. Nat Genet. mai 2001;28(1):53‑7.

20. Atcha FA, Munguia JE, Li TWH, Hovanes K, Waterman ML. A new beta-catenin-dependent activation domain in T cell factor. J Biol Chem. 2 mai 2003;278(18):16169‑75.

21. Yamamoto Y, Sakamoto M, Fujii G, Tsuiji H, Kenetaka K, Asaka M, et al. Overexpression of orphan G-protein-coupled receptor, Gpr49, in human hepatocellular carcinomas with beta-catenin mutations. Hepatology. mars 2003;37(3):528‑33.

22. He TC, Sparks AB, Rago C, Hermeking H, Zawel L, da Costa LT, et al. Identification of c-MYC as a target of the APC pathway. Science. 4 sept 1998;281(5382):1509‑12.

23. Koch A, Waha A, Hartmann W, Hrychyk A, Schüller U, Waha A, et al. Elevated expression of Wnt antagonists is a common event in hepatoblastomas. Clin Cancer Res. 15 juin 2005;11(12):4295‑304.

24. Torisu Y, Watanabe A, Nonaka A, Midorikawa Y, Makuuchi M, Shimamura T, et al. Human homolog of NOTUM, overexpressed in hepatocellular carcinoma, is regulated transcriptionally by beta-catenin/TCF. Cancer Sci. juin 2008;99(6):1139‑46.

25. Chen J, Rajasekaran M, Xia H, Zhang X, Kong SN, Sekar K, et al. The microtubule-associated protein PRC1 promotes early recurrence of hepatocellular carcinoma in association with the Wnt/β-catenin signalling pathway. Gut. sept 2016;65(9):1522‑34.

26. Torre C, Benhamouche S, Mitchell C, Godard C, Veber P, Letourneur F, et al. The transforming growth factor-α and cyclin D1 genes are direct targets of β-catenin signaling in hepatocyte proliferation. J Hepatol. juill 2011;55(1):86‑95.

27. Blache P, van de Wetering M, Duluc I, Domon C, Berta P, Freund JN, et al. SOX9 is an intestine crypt transcription factor, is regulated by the Wnt pathway, and represses the CDX2 and MUC2 genes. J Cell Biol. 5 juill 2004;166(1):37‑47.

28. Kawai T, Yasuchika K, Ishii T, Miyauchi Y, Kojima H, Yamaoka R, et al. SOX9 is a novel cancer stem cell marker surrogated by osteopontin in human hepatocellular carcinoma. Sci Rep. 26 juill 2016;6:30489.

29. Leung CON, Mak WN, Kai AKL, Chan KS, Lee TKW, Ng IOL, et al. Sox9 confers stemness properties in hepatocellular carcinoma through Frizzled-7 mediated Wnt/β-catenin signaling. Oncotarget. 17 mai 2016;7(20):29371‑86.

30. Liu S, Yeh TH, Singh VP, Shiva S, Krauland L, Li H, et al. β-catenin is essential for ethanol metabolism and protection against alcohol-mediated liver steatosis in mice. Hepatology. mars 2012;55(3):931‑40.

31. Benhamouche S, Decaens T, Godard C, Chambrey R, Rickman DS, Moinard C, et al. Apc tumor suppressor gene is the « zonation-keeper » of mouse liver. Developmental cell. juin 2006;10(6):759‑70.

32. Yan BC, Gong C, Song J, Krausz T, Tretiakova M, Hyjek E, et al. Arginase-1: a new immunohistochemical marker of hepatocytes and hepatocellular neoplasms. Am J Surg Pathol. août 2010;34(8):1147‑54.

33. Braeuning A, Ittrich C, Köhle C, Hailfinger S, Bonin M, Buchmann A, et al. Differential gene expression in periportal and perivenous mouse hepatocytes. FEBS J. nov 2006;273(22):5051‑61.

34. Gougelet A, Torre C, Veber P, Sartor C, Bachelot L, Denechaud PD, et al. T-cell factor 4 and beta-catenin chromatin occupancies pattern zonal liver metabolism in mice. Hepatology. juin 2014;59(6):2344‑57.

35. Cadoret A, Ovejero C, Terris B, Souil E, Levy L, Lamers WH, et al. New targets of beta-catenin signaling in the liver are involved in the glutamine metabolism. Oncogene. 28 nov 2002;21(54):8293‑301.

36. Ovejero C, Cavard C, Périanin A, Hakvoort T, Vermeulen J, Godard C, et al. Identification of the leukocyte cell-derived chemotaxin 2 as a direct target gene of beta-catenin in the liver. Hepatology. juill 2004;40(1):167‑76.

37. Anson M, Crain-Denoyelle AM, Baud V, Chereau F, Gougelet A, Terris B, et al. Oncogenic β-catenin triggers an inflammatory response that determines the aggressiveness of hepatocellular carcinoma in mice. J Clin Invest. févr 2012;122(2):586‑99.

38. Charawi S, Just PA, Savall M, Abitbol S, Traore M, Metzger N, et al. LKB1 signaling is activated in CTNNB1-mutated HCC and positively regulates β-catenin-dependent CTNNB1-mutated HCC. J Pathol. avr 2019;247(4):435‑43.

39. Cavard C, Terris B, Grimber G, Christa L, Audard V, Radenen-Bussiere B, et al. Overexpression of regenerating islet-derived 1 alpha and 3 alpha genes in human primary liver tumors with beta-catenin mutations. Oncogene. 26 janv 2006;25(4):599‑608.

40. Boyault S, Rickman DS, de Reynies A, Balabaud C, Rebouissou S, Jeannot E, et al. Transcriptome classification of HCC is related to gene alterations and to new therapeutic targets. Hepatology (Baltimore, Md. janv 2007;45(1):42‑52.

41. Renard CA, Labalette C, Armengol C, Cougot D, Wei Y, Cairo S, et al. Tbx3 is a downstream target of the Wnt/beta-catenin pathway and a critical mediator of beta-catenin survival functions in liver cancer. Cancer Res. 1 févr 2007;67(3):901‑10.
