# Supplementary material for: Specific features of ß-catenin-mutated hepatocellular carcinomas
Source: Br J Cancer. 2024 Sep 11;131(12):1871–80. doi: 10.1038/s41416-024-02849-7 (PMC11628615; doi:10.1038/s41416-024-02849-7)
Supplement: Supplementary file 3 — Supplemental Table II [file 41416_2024_2849_MOESM3_ESM.docx]

**Supplemental Table II: Clinical trials using anti-ß-catenin strategies**

| **Drug** | **Target** | **Cancer type** | **Phase** | **Clinical trial** | **Status** |
| --- | --- | --- | --- | --- | --- |
| *Liver-related diseases* | | | | | |
| PRI-724 / OP-724 / Foscenvivint | CBP/ß-catenin antagonist | Liver Cirrhosis,  Hepatitis B, C, Liver Cirrhoses  Hepatitis C Virus-infected Cirrhosis  Liver Cirrhosis  Primary Biliary Cholangitis (PBC)  Liver Cirrhosis, Biliary | I  I, IIa  I  II  I | NCT04688034  NCT03620474  NCT02195440  NCT06144086  NCT04047160 | Completed  Completed  Completed  Recruiting  Completed |
| PRI-724 | CBP/ß-catenin antagonist | Advanced Solid Tumors | I | NCT01302405 | Terminated (closing due to low enrollment) |
| E7386 | CBP/ß-catenin antagonist | Colorectal Neoplasms  Gastrointestinal Tumors  Solid Neoplasms | I | NCT03833700 | Active, Not Recruiting |
| E7386 | CBP/ß-catenin antagonist | Advanced Neoplasms | I | NCT03264664 | Active, Not Recruiting |
| E7386 with Lenvatinib | CBP/ß-catenin antagonist | Carcinoma, Hepatocellular  Colorectal Neoplasms  Endometrial Neoplasms  Liver Neoplasms  Neoplasms | Ib | NCT04008797 | Recruiting |
| E7386 with Pembrolizumab | CBP/ß-catenin antagonist | Carcinoma, Hepatocellular  Colorectal Neoplasms  Melanoma | Ib, II | NCT05091346 | Active, Not Recruiting |
| Tegavivint | Inhibitor of TBL1/ß-catenin complex | Colorectal Carcinoma  Endometrial Carcinoma  Melanoma  Neuroblastoma  Ovarian Carcinoma  Pancreatic Ductal Adenocarcinoma  Recurrent Desmoid Fibromatosis  Recurrent Ewing Sarcoma  Recurrent Hepatoblastoma  Recurrent Hepatocellular Carcinoma  Recurrent Malignant Solid Neoplasm  Recurrent Non-Hodgkin Lymphoma  Recurrent Osteosarcoma  Refractory Desmoid Fibromatosis  Refractory Ewing Sarcoma  Refractory Hepatoblastoma  Refractory Hepatocellular Carcinoma  Refractory Malignant Solid Neoplasm  Refractory Non-Hodgkin Lymphoma  Refractory Osteosarcoma  Solid Pseudopapillary Neoplasm of the Pancreas  Wilms Tumor | I, II | NCT04851119 | Recruiting |
| Tegavivint (alone and with pembrolizumab) | Inhibitor of TBL1/ß-catenin complex | Advanced Hepatocellular Carcinoma | I | NCT05797805 | Recruiting |
| *Other diseases* | | | | | |
| PRI-724 | CBP-B-catenin antagonist | Acute Myeloid Leukemia  Chronic Myeloid Leukemia |  | NCT010606579 | Completed |
| PRI-724 | CBP-B-catenin antagonist | Advanced Pancreatic Cancer  Metastatic Pancreatic Cancer  Pancreatic Adenocarcinoma |  | NCT01764477 | Completed |
| PRI-724 with bevacizumab | CBP-B-catenin antagonist | Colorectal Adenocarcinoma  Stage IVA Colorectal Cancer  Stage IVB Colorectal Cancer |  | NCT02413853 | Withdrawn |
| Tegavivint | Inhibitor of TBL1/ß-catenin complex | Relapsed or Refractory Large B-Cell Lymphoma | I | NCT05755087 | Recruiting |
| Tegavivint | Inhibitor of TBL1/ß-catenin complex | Relapsed or Refractory Leukemia | I | NCT04874480 | Recruiting |
| Tegavivint with Osimertinib | Inhibitor of TBL1/ß-catenin complex | Metastatic EGFR-Mutant Non-small Cell Lung Cancer | Ib | NCT04780568 | Recruiting |
